# How Does Age at Onset Influence the Outcome of Autoimmune Diseases?

**DOI:** 10.1155/2012/251730

**Published:** 2011-12-13

**Authors:** Manuel J. Amador-Patarroyo, Alberto Rodriguez-Rodriguez, Gladis Montoya-Ortiz

**Affiliations:** Center for Autoimmune Diseases Research (CREA), School of Medicine and Health Sciences, Universidad del Rosario, Carrera 24 # 63-C-69, Bogotá, Colombia

## Abstract

The age at onset refers to the time period at which an individual experiences the first symptoms of a disease. In autoimmune diseases (ADs), these symptoms can be subtle but are very relevant for diagnosis. They can appear during childhood, adulthood or late in life and may vary depending on the age at onset. Variables like mortality and morbidity and the role of genes will be reviewed with a focus on the major autoimmune disorders, namely, systemic lupus erythematosus (SLE), rheumatoid arthritis (RA), multiple sclerosis (MS), type 1 diabetes mellitus (T1D), Sjögren's syndrome, and autoimmune thyroiditis (AITD). Early age at onset is a worst prognostic factor for some ADs (i.e., SLE and T1D), while for others it does not have a significant influence on the course of disease (i.e., SS) or no unanimous consensus exists (i.e., RA and MS).

## 1. Introduction

Autoimmune diseases (ADs) affect approximately 5% of the world population [1, 2]. The age at onset varies widely depending on the disease. For example, sixty-five percent of patients with systemic lupus erythematosus (SLE) start manifesting their symptoms between ages 16 and 55 [3]. Another 20 percent manifest them before age 16 and the remaining 15 percent after age 55 [4]. Rheumatoid arthritis (RA) can begin at any age but has its peak between ages 30 and 55 [5]. Juvenile idiopathic arthritis (JIA) is a term used to describe the autoimmune, inflammatory joint condition that develops in children. Another prevalent AD is Sjögren's syndrome (SS) which is considered to be more prevalent in women between ages 45 and 50 [6]. Multiple Sclerosis (MS) usually appears between ages 20 and 40, and it is very rare during adolescence [7]. Type 1 diabetes mellitus (T1D) is considered a childhood and adolescent disease with two peaks of onset, one between ages 5 and 9 and a second between ages 10 and 14 [8]. On the other hand, an adult onset would be considered to be in a range of 25–61 years old [9]. Finally, autoimmune thyroiditis (AITD) is thought to be a disease that can appear in childhood but is more prevalent during adulthood [10]. Herein, we analytically reviewed the effect of age at onset on the most prevalent ADs, their clinical differences (Table 1), and their genetic and immunologic relationships (Table 2).

## 2. Systemic Lupus Erythematosus

SLE is a chronic AD that affects individuals of every age. It is more common in adults, but it may be diagnosed during childhood as well [11]. Childhood disease onset is characterized by a high degree of morbidity compared with adult SLE populations [12]. It is associated with a higher mean in the Systemic Lupus Erythematosus Disease Activity Index (SLEDAI) scores at presentation. The patients have a higher frequency of renal disease, malar rashes, pericarditis, hepatosplenomegaly, and hematologic alterations [13]. In treatment, they usually have a higher use of prednisone and the need for additional immunosuppressive therapies [14]. Patients at this age are susceptible to a longer lifetime of damage from the disease flares and the treatment side effects and a mortality rate that is 2 times higher [11, 12]. Other associations found in childhood onset are growth failure, delayed puberty, and fibromyalgia. In contrast, adult onset patients are more likely to develop pulmonary disease [11] and may have an increased rate of simultaneously developing another AD such as SS [15]. Additionally, Webb et al. showed that childhood onset had higher odds of presenting proteinuria, haemolytic anaemia, arthritis, leucopenia, and anti-dsDNA antibody. They also showed that early adult onset (≤50 years) had greater odds of having proteinuria, cellular casts, seizures, anti-nRNP antibody, anti-Sm antibody, and anti-dsDNA antibody than late-adult-onset patients (>50 years) [16]. Hersh et al. stated that rates for clotting disorders, myocardial infarction, and neurological disorders (e.g., seizures) are similar in both of them [11]. In both cases, better disease outcome can be achieved if an early diagnosis is made by a better recognition of age-specific manifestations and the use of a good treatment algorithm [15].

As other ADs, SLE is a complex disease in which several polymorphic genes and environmental factors over time influence the onset and course of disease. Among the genes found to be associated with SLE, *MBL2* gene has been suggested to influence the age at onset [17]. Webb et al. showed that odds of developing SLE during childhood (<18 years) increased by 48% per risk allele in Gullah people with SLE and 25% in African-American patients. No association between the number of SLE risk alleles and age at onset in Hispanic patients and European American was found [16].

## 3. Rheumatoid Arthritis and Juvenile Idiopathic Arthritis

RA is a chronic, systemic, and destructive inflammatory AD that involves both small and large diarthrodial joints. It usually develops in middle-aged adults but may also appear during childhood or late in life [18]. Patients who are diagnosed between ages 16 and 65 are considered young onset and after 65, late onset with each of them having different semiologic characteristics. Pease et al. found that late-onset patients had longer stiffness in the morning [19]. In contrast, Deal et al. reported no difference in morning stiffness between young- and late-onset patients [5]. It has been reported that older patients have more acute onset in both large (specially the shoulders) and small joints and usually present polymyalgia rheumatica-like symptoms [20]. Turkcapar et al. reported that proximal interphalangeal, metacarpophalangeal, elbow, metatarsophalangeal, and ankle joints are more associated with young-onset AR. Classical hand deformities, interstitial lung disease, and SS presented less commonly at late onset. However, these patients can present more constitutional features like weight loss, myalgia, rheumatic nodules, and neuropathy [21]. Anti-CCP seropositivity and elevated inflammatory markers at onset are associated with poor radiological outcome in both early and late onset [22, 23]. Studies differ when talking about prognosis and report favorable, similar, or worse outcome. Most of them conclude that treatment should be instituted equally on both.

Juvenile idiopathic arthritis (JIA) is a term that describes a group of disorders that share the clinical manifestation of chronic joint inflammation from unknown causes that begins before 16 years of age [24]. It is divided into seven groups being polyarticular JIA (PJIA), the one most similar to RA and the most related to rheumatoid factor positivity [25]. PJIA is defined by the presence of more than four affected joints during the first six months of illness. There is a bimodal distribution of the age at onset: from 2 to 5 years and 10 to 14. Children with polyarticular disease with onset after 10 years of age are divided into negative rheumatoid factor (RF) polyarthritis and positive RF polyarthritis. Positive RF polyarthritis tends to be associated with anti-CCP antibodies and a more severe disease than their adult counterpart [26]. They usually have a rapid onset of inflammation in multiple joints, especially hands, wrists, elbows, and feet. A big difference between it and the adult form of the disease is the effect of the disease on their growing skeleton that can lead to growth retardation or accelerated growth of an affected joint [24].

The HLA-DRB1 gene has been associated with RA susceptibility, especially with those alleles carrying the shared epitope [27]. Anti-CCP antibodies can be detected at early disease stages and may be used as indicators of RA progression and prognosis [28]. Diaz et al. reported that anti-CCP antibodies and the HLA-DRB1 DERAA sequence influence the age at onset of RA [29]. This finding may be useful to predict early RA onset in genetically predisposed patients [29]. In JIA, RF-positive polyarthritis with anti-CCP antibodies is associated with the presence of HLA–DR4 alleles and an aggressive disease course [26].

## 4. Sjögren's Syndrome

SS is a chronic AD characterized by xerophthalmia and xerostomia caused by a progressive lymphocytic and plasma cell infiltration in the exocrine glands and may also have systemic involvement. It is very rare during childhood [30]. Drosos et al. showed that recurrent parotid gland enlargement was more common in early age onset while sicca symptoms were more common in adults [31]. Ostuni et al. reported that the clinical manifestations were similar but extraglandular manifestations were milder [32]. Anaya et al. implied that the clinical symptoms in children do not fulfill the classical diagnostic criteria which are successfully applied to adults [33]. Botsios et al. found that young, adult and elderly patients had similar sensitivity of diagnostic test positivity [34]. In contrast, Nakamura et al. showed the prevalence of anti-muscarinic 3 receptor (anti-M3R) antibodies is higher in early-onset SS than in late-onset SS patients [35]. However, this findings warrant further replication and confirmation.

HLA-DRB1*0301-DQB1*0201 haplotype has been associated with SS [36]. No age at onset relationship was found.

## 5. Type 1 Diabetes

T1D is a chronic AD resulting from progressive destruction of the pancreatic B cells. B-cell damage may be induced at any age [37]. Childhood- and late-onset patients are characterized by symptoms like polydipsia, polyuria, and weight loss, but younger patients suffer more from diabetic ketoacidosis and ketosis as the initial presentation [38, 39]. Studies indicate that late-onset patients have better preserved B-cell function than early-onset patients. They are also characterized by a longer symptomatic period before diagnosis and a lower frequency of insulin autoantibodies and HLA class II susceptibility alleles [40, 41]. Adult onset can be associated with milder signs of metabolic decompensation and a lower glycated hemoglobin level at diagnosis [39, 42].

Associated HLA-Class II alleles for development of the disease are identified. DRB1*0301-DQA1*0501-DQB1*0201 is considered risk alleles and DRB1*1301-DQB1*0603-DQB1*0602, protective alleles [43]. The high-risk HLA DRB1*0301 and DQB1*02/*0302 alleles are commonly associated with young onset as well as absence of DQB1*0602. Adult patients carry high-risk DQB1*02/0302 less frequently [39, 42, 44]. The *PTPN22* C1858T polymorphism has also been associated with higher risk for development of ADs, specifically T1D, RA, SLE, and Graves disease. This polymorphism could be associated with development of T1D at an early age [45]. Lee et al. showed that *STAT4* polymorphism is associated with early-onset T1D and not with late onset in Asian population and also suggested a dosage effect of risk alleles on the age of onset of disease [46].

## 6. Multiple Sclerosis

Age at onset of MS as in most of ADs is defined as the age when the first symptoms appear, although the disease process may have begun earlier [47]. Duquette et al. reported an early-onset prevalence of 2.7% with respect to the entire MS population [48]. Studies are consistent with the idea that, in early onset, mild and severe disability levels are reached after a longer time than in the case of adult onset. However, these disability levels are also reached at a lower age in comparison with adult cases. This can be translated into more disability in early-onset patients than their corresponding adult counterpart at the same age. Bad prognostic factors for a worse outcome include disability in the first year, high relapse rate, short interattack intervals, the relapsing progressive course, or a shift of the progressive phase [7, 49–51]. Deryck et al. showed specific clinical characteristics in patients with early onset such as mainly relapsing remitting disease onset and frequent presentation with brainstem-cerebellar dysfunction, pyramidal symptom, and optic neuritis [52]. Kis et al. showed that primary progressive course and motor symptoms are more characteristic of late-onset patients [53]. Trojano et al. state that current age, together with duration of disease and apart from age at onset, influences MS progression [54].

Associated HLA-class alleles of the disease are also identified. DRB1*1501 and DQB1*0602 were found to be high risk factors for MS [55]. No onset relationship was described, but high risk factor allele DQB1*0602 is thought to be protective in T1D.

## 7. Autoimmune Thyroiditis

AITD is an inflammatory state of the thyroid gland that results from interaction between genetic and environmental factors. Hashimoto thyroiditis is the most frequent form of chronic autoimmune thyroiditis. This is the most common cause of hypothyroidism in children [56]. Some of its manifestations at this age are effects on growth, bad school performance, bradykinesia, and delayed pubertal development. A goiter is the main symptom that indicates AITD. Patients that receive proper treatment at this age will probably experience normal growth and puberty [57, 58]. Both young and adult onset patients may show symptoms of lethargy, intolerance to cold, constipation, dry skin, brittle hair, and muscle pain. Late-onset hypothyroidism, once it begins, is permanent but, in some young onset patients and postpartum women, it is often transient [10]. Young patients with other ADs are at increased risk of having AITD. A good example is T1D. Riley et al. showed that approximately 20 percent of T1D patients have high serum antithyroid antibody concentrations and 5 percent have abnormalities in thyroid function [59]. That is why they have to be screened annually.

Genetic susceptibility is linked to the HLA DR3 group [57] and to polymorphisms at *CTLA-4* gene, among others. Cho et al. suggested that coexistence of HLA-B*46 and HLA-Cw*01 may be a genetic marker for early-onset AITD in Koreans [60].

## 8. Conclusions

Age at onset varies among ADs and so do their manifestations. Some of them, for example, MS and SS, are rare during childhood but others such as T1D primarily occur during this period. Early age at onset cannot always be associated with a worse prognosis. Early age at onset is a worst prognostic factor for some ADs (i.e., SLE and T1D), while for others it does not have a significant influence on the course of disease (i.e., SS) or no unanimous consensus exists (i.e., RA and MS). Knowledge of the early-age symptoms will help physicians to provide better treatment which, coupled with educational and transition support, might improve outcome. Understanding of genetic influences and association studies between diseases are required to determine the role of genes in age at onset. Studies with a larger number of people with nontypical ages at onset would bring further insights.

## Figures and Tables

**Table 1:** Clinical differences between early and adult onset.

AD	Early onset	Late onset	
SLE	Higher degree of morbidity [12] Higher (SLEDAI) score at presentation Higher incidence of renal disease Malar rashes Pericarditis Hepatosplenomegaly Hematologic alterations (Leucopenia) Higher use of prednisone Additional immunosuppressive therapies Greater lifetime of damages from the disease flares and the treatment side effects [13] 2 times higher mortality rate Growth failure Delayed puberty Fibromyalgia [11] Higher odds of presenting proteinuria Haemolytic anaemia Arthritis [16]	Increased rate of pulmonary disease [11] Increased rate of simultaneously developing another AD [12]	

RA	Proximal Interphalangeal, metacarpophalangeal, elbow, metatarsophalangeal, and ankle joints Classical rheumatoid hand deformities Interstitial lung disease Associated SS [21] Shorter morning stiffness [19]	Acute onset in large and small joints (specially shoulders) PMR-like symptoms [20] Constitutional features Weight loss Myalgia Rheumatic nodules Neuropathy [21] Longer morning stiffness [19]	Positive RF PJIA* Rapid onset of inflammation in multiple joints Proximal interphalangeal, metacarpophalangeal, elbow, metatarsophalangeal, and ankle joints Effects of the disease in a growing skeleton: Growth retardation Accelerated growth of an affected joint [24]

SS	Recurrent parotid gland enlargement Milder extraglandular manifestations [32]	Sicca symptoms [31]	

T1D	Ketosis and ketoacidosis Higher mean glycated hemoglobin [38, 40]	Better preserved B-cell function Longer symptomatic period before diagnosis Less frequency of insulin autoantibodies and HLA class II susceptibility alleles [40, 41] Milder signs of metabolic decompensation and a lower glycated hemoglobin level at diagnosis [39, 42]	

MS	Mainly relapsing remitting disease onset Frequent presentation with brainstem-cerebellar dysfunction Pyramidal symptom Optic neuritis [52]	Primary progressive course Motor symptoms are predominant	

AITD	Often transient [10] Delayed growth Bradykinesia Delayed pubertal development [57, 58]	Permanent [10]	

AD: autoimmune disease; RA: rheumatoid arthritis; SS: Sjögren's syndrome; T1D: type 1 diabetes; MS: multiple sclerosis; AITD: autoimmune thyroiditis; PJIA: polyarticular juvenile idiopathic arthritis; RF: rheumatoid factor.

*In RA, early age at onset is considered ≥16 years old. Positive RF PJIA is considered a comparable disease with a childhood onset (<16 years old).

**Table 2:** Genetic and immunological factors related to age at onset.

Autoimmune disease	Population	Immunologic	Genetic
SLE early onset*	African Americans and Gullah		Odds of developing the disease increased by 48% per risk allele in Gullah patients and 25% in African-American patients [16]
	Caucasians Hispanics African Americans and Gullah	Higher odds of presenting Anti-dsDNA antibody [16]	
	Caucasians		Association with *MBL2* gene [17]

Positive RF PJIA	Caucasians	Higher frequency of Anti-CCP antibodies	HLA-DR4 alleles [26]

RA early onset	Hispanics	Higher frequency of Anti-CCP antibodies	HLA-DRB1 *DERAA* sequence [29]

SS early onset	Asians	Higher prevalence of anti-M3R Antibodies [35]	

T1D early Onset	Caucasians		Association with high Risk HLA DQB1*02/*0302 [44] Association with * PTPN22 *C1858T [45]
	African Americans		Association with absence of DQB1*0602 and increase in DQB1*0201 [42]
	Asians		Association with STAT4 polymorphism [46]

T1D late onset	Caucasians	Less frequency of insulin autoantibodies	

MS susceptibility	Hispanics		High risk DQB1*0602 susceptibility allele is the same that protects in T1D [55]

AITD early onset	Asians		Coexistence of HLA-B*46 and HLA-Cw*01 [60]

AD: autoimmune disease; RA: rheumatoid arthritis; SLE: systemic lupus erythematosus; SS: Sjögren's syndrome; M3R: muscarinic 3 receptor; T1D: type 1 diabetes; MS: multiple sclerosis; AITD: autoimmune thyroiditis; PJIA: polyarticular juvenile idiopathic arthritis; RF: rheumatoid factor.

*Defined as ≤20 years old.
